# Safety of Renal Biopsy by Physicians with Short Nephrology Experience

**DOI:** 10.3390/healthcare9040474

**Published:** 2021-04-16

**Authors:** Kenta Torigoe, Kumiko Muta, Kiyokazu Tsuji, Ayuko Yamashita, Shinichi Abe, Yuki Ota, Hiroshi Mukae, Tomoya Nishino

**Affiliations:** 1 Department of Nephrology, Nagasaki University Hospital, Nagasaki 852-8501, Japan; ktorigoe@nagasaki-u.ac.jp (K.T.); tsuji-kiyo@nagasaki-u.ac.jp (K.T.); ayamashita@nagasaki-u.ac.jp (A.Y.); shinichiabe4227@gmail.com (S.A.); yukiota@hospital.sasebo.nagasaki.jp (Y.O.); tnishino@nagasaki-u.ac.jp (T.N.); 2 Department of Respiratory Medicine, Nagasaki University Graduate School of Biomedical Sciences, Nagasaki 852-8501, Japan; hmukae@nagasaki-u.ac.jp

**Keywords:** renal biopsy, clinical experience, complications, nephrologist, supervision

## Abstract

Percutaneous renal biopsy is an essential tool for diagnosing various renal diseases; however, little is known about whether renal biopsy performed by physicians with short nephrology experience is safe in Japan. This study included 238 patients who underwent percutaneous renal biopsy between April 2017 and September 2020. We retrospectively analyzed the frequency of post-renal biopsy complications (hemoglobin decrease of ≥10%, hypotension, blood transfusion, renal artery embolization, nephrectomy and death) and compared their incidence among physicians with varied experience in nephrology. After renal biopsy, a hemoglobin decrease of ≥10%, hypotension and transfusion occurred in 13.1%, 3.8% and 0.8% of patients, respectively. There were no cases of post-biopsy renal artery embolism, nephrectomy, or death. The composite complication rate was 16.0%. The incidence of post-biopsy complications was similar between physicians with ≥3 years and <3 years of clinical nephrology experience (12.5% vs. 16.8%, *p* = 0.64). Furthermore, the post-biopsy composite complication rates were similar between physicians with ≥6 months and <6 months of clinical nephrology experience (16.3% vs. 15.6%, *p* > 0.99). Under attending nephrologist supervision, a physician with short clinical nephrology experience can safely perform renal biopsy.

## 1. Introduction

Percutaneous renal biopsy is an important procedure that contributes to diagnosis, prognosis prediction and treatment plan decisions in various renal diseases. Since Iversen and Brun’s first report of percutaneous renal biopsy in 1951 [1], continuous technical developments have been made and, at present, ultrasound-guided needle biopsy is widely performed. The major complication of renal biopsy is bleeding, which is often relieved by conservative management (e.g., bedrest, antihemorrhagic agent and fluid replacement therapy); however, severe cases of bleeding do occur and may require blood transfusions, renal artery embolization, or nephrectomy [2]. Numerous factors, such as older age, female sex, anemia, biopsy for acute kidney injury, use of 14-gauge needles and high serum creatinine levels, are associated with the risk of bleeding after renal biopsy [3,4]. Most complications occur within 8 h after renal biopsy [4]. Given that renal biopsy is a diagnostic test rather than a therapeutic procedure, efforts must be made to avoid complications. Clinicians need to evaluate the risk factors for complications before renal biopsy and carefully check the patient’s condition immediately after the biopsy.

Several procedures and surgeries, including renal biopsy, require sufficient training and experience to master. Therefore, when certain procedures are performed by trainees, the incidence of complications may increase. For example, in laparoscopic gastric surgery, involvement of trainees during the first 6 months of their fellowship is reportedly associated with an increased rate of complications [5]. Conversely, reports from the United States and Italy did not find a negative effect on safety when renal biopsy is performed by nephrology trainees [6,7].

The educational and clinical practice environments are not always the same throughout the world. For example, in the United States, renal biopsies are performed by both nephrologists and radiologists [8], but in Japan, they are performed mainly by nephrologists [9]. Nephrology trainees may be able to safely perform renal biopsy in settings where nephrologists are closely involved in the renal biopsy procedure; as such, it is possible that, like trainees in other countries, nephrology trainees in Japan can also perform renal biopsy safely. However, to our knowledge, no study has reported whether renal biopsy performed by physicians with short clinical experience in nephrology is safe in Japan. In this study, we investigated the safety of percutaneous renal biopsy performed at a Japanese teaching hospital by physicians with short clinical experience in nephrology.

## 2. Materials and Methods

### 2.1. Ethics Statement

All procedures performed in studies involving human participants were in accordance with the ethical standards of the institutional and/or national research committee at which the studies were conducted (Institutional Review Board approval number, 21021519) and with the 1964 Declaration of Helsinki and its later amendments or comparable ethical standards. Informed consent was obtained in the form of an opt-out on our institution’s website.

### 2.2. Patient Selection

In this study, we retrospectively reviewed the data of 238 patients who underwent percutaneous renal biopsy at Nagasaki University Hospital Department of Nephrology between April 2017 and September 2020. Cases of percutaneous renal biopsies for transplanted kidneys were excluded because the procedure and the frequency of complications differ between transplanted renal biopsy and native renal biopsy [10].

### 2.3. Data Collection

Data measured just prior to renal biopsy were collected as patient baseline data. If a red blood cell transfusion was performed before the renal biopsy, the expected increase in hemoglobin (Hb) after the transfusion (dividing the dosed Hb amount [g] by the circulating plasma volume (dL)) was added to the Hb level before the renal biopsy. All renal biopsies were performed by members of the Japanese Society of Nephrology. For each physician, the months or years of experience in clinical nephrology were recorded.

Post-renal biopsy blood transfusion, hypotension (due to the vasovagal response or bleeding), renal artery embolization, nephrectomy and death were recorded as complications. Based on a previous study, we also recorded the number of cases in which Hb decreased by ≥10% after renal biopsy [11]. Furthermore, as an evaluation of biopsy quality, the number of glomeruli collected was counted.

### 2.4. Renal Biopsy Procedure

The indications and risk assessment for complications for all biopsies were approved by an attending nephrologist (board-certified nephrologist of the Japanese Society of Nephrology or educator of the Japanese Society of Nephrology). If the patient was on anticoagulants or antiplatelet therapy, the drugs were stopped and heparin bridging was performed. All renal biopsies were ultrasound-guided needle biopsies performed in a hospitalized setting according to local practice. Briefly, patients were placed in the prone position with an electrocardiogram monitor, non-invasive blood pressure monitor and pulse oximeter. Patients underwent cannulated peripheral venous catheter insertion and injection of an antihemorrhagic agent before the biopsy. Except when an attending nephrologist performed the renal biopsy, the attending nephrologist always supervised and checked whether the biopsy was performed properly. The puncture site was the lower pole of the right or left kidney. After disinfecting the patient’s skin, local anesthesia was instituted with 1% xylocaine and the biopsy was performed using an automated biopsy gun loaded with a 16-gauge needle. Real-time ultrasound guidance and biopsy needle puncture were performed by a single nephrologist. The biopsies were terminated when a sufficient specimen was collected or when continuation was judged to be dangerous. The puncture site was pressed firmly for 10 min immediately after the biopsy. Biopsies were mainly performed in the early afternoon, after which the patient was instructed to stay in the prone position for the next hour and was kept in the supine position in the bed until the following day. If there were no adverse events or no evidence of progression of anemia in the complete blood cell count, the patients were permitted to ambulate.

### 2.5. Statistical Analysis

Categorical variables are expressed as the number (%). Continuous variables are expressed as the mean ± standard deviation and non-normally distributed data are expressed as the median and interquartile range. To become a board-certified nephrologist of the Japanese Society of Nephrology, at least 3 years of clinical nephrology experience is required. Therefore, we compared the patient characteristics and post-biopsy data between physicians with ≥3 years and <3 years of clinical nephrology experience. Furthermore, the same analysis was performed between physicians with ≥6 months and <6 months of clinical nephrology experience. Comparisons of nominal variables between the two groups were performed using Fisher’s exact test or Pearson’s chi-squared test, while the Wilcoxon rank sum test was performed when comparing continuous variables. Furthermore, we defined a post-renal biopsy Hb decrease of ≥10%; hypotension; death; and intervention requirement, such as blood transfusion, renal artery embolization and nephrectomy, as the post-renal biopsy composite complication. Logistic regression analysis was performed to investigate the factors associated with the post-renal biopsy composite complication. Independent variables were the physicians’ clinical nephrology experience and other risk factors for post-renal biopsy complications; we selected age, sex, pre-biopsy Hb and estimated glomerular filtration rate (eGFR) based on previous studies [3,4,12]. Statistical analyses were performed using JMP version 13 software (SAS Institute Inc., Cary, NC, USA). A *p*-value of less than 0.05 was considered indicative of statistical significance.

## 3. Results

### 3.1. Patient Characteristics

Patient characteristics are shown in Table 1. The mean age of the patients was 54.1 ± 18.1 years and 127 patients (53.4%) were men. The mean pre-biopsy Hb and eGFR were 12.2 ± 2.2 g/dL and 54.9 ± 27.9 mL/min/1.73 m^2^, respectively. Renal biopsies were performed by 18 nephrologists and the mean number of glomeruli obtained was 20.4 ± 11.1. After the renal biopsy, an Hb decline of ≥10%, hypotension and transfusion occurred in 13.1%, 3.8% and 0.8% of patients, respectively. There were no cases of post-biopsy renal artery embolism, nephrectomy or death. The composite complication rate was 16.0%.

### 3.2. Comparisons According to Nephrology Experience

Next, we compared the post-biopsy complication rate between physicians with ≥3 years and <3 years of clinical nephrology experience. As shown in Table 2, the post-biopsy composite complication rate was similar between the groups. Since the quality of the tissue sample is a critical issue and the number of glomeruli obtained by renal biopsy is an indicator of quality, we compared the number of glomeruli obtained between the two groups. The results showed similar numbers in both groups.

Procedural complications may occur when biopsies are performed by trainees in the preliminary stages. Therefore, we compared the post-biopsy composite complication rate between physicians with ≥6 months and <6 months of clinical nephrology experience. As shown in Table 3, the post-biopsy composite complication rate was similar between the groups.

### 3.3. Clinical Nephrology Experience Is Not Associated with Post-biopsy Complications

We subsequently performed a logistic regression analysis to investigate the risk factors for the composite complication after renal biopsy. As shown in Table 4, the univariate analysis revealed that age, sex, pre-biopsy Hb, eGFR and years of clinical nephrology experience were not associated with the composite complication. Similar results were found in the multivariate analysis, including the result obtained for years of clinical nephrology experience. These results suggest that renal biopsy can be safely performed by physicians with short experience in clinical nephrology.

## 4. Discussion

In this study, we showed that, under attending nephrologist supervision, a physician with short experience in clinical nephrology can safely perform renal biopsy.

Previous studies had reported that nephrology fellows could perform renal biopsies as safely as attending nephrologists [6,7]. However, the safety of renal biopsy may not be equivalent worldwide because of differences in medical practices (e.g., specialty of the operating physician or hospitalization). A nationwide survey in Japan reported that a blood transfusion after renal biopsy was required in about 0.7% of cases [9]. Herein, 0.5% of patients required a blood transfusion after renal biopsy performed by physicians with <3 years of clinical nephrology experience (Table 3). This finding suggests that the renal biopsies performed by physicians with short clinical nephrology experience in this study achieved similar safety levels as the nationwide standards in Japan. Previous studies investigating renal biopsy safety mainly focused on whether or not the performer was a nephrology trainee [6,7]. However, it is unclear whether renal biopsy can be performed safely by trainees in the early stages of their training. This study showed that renal biopsy can be performed safely even by physicians with <6 months of clinical nephrology experience. Furthermore, some of the physicians involved were performing renal biopsy for the first time and yet were able to complete it safely. This result may be helpful at the early stage for nephrology trainees in not only Japan, but also other countries.

In the present study, renal biopsies were supervised by an attending nephrologist when the performer was a trainee. Supervision by an attending nephrologist is important for training the fellows [13] and ensuring the safety of the procedure [14,15]. In Japan, renal biopsy is mainly performed by nephrologists [16], which suggests that attending nephrologists have extensive experience in renal biopsy and can provide appropriate supervision. Similarly, previous studies from the United States and Italy showed that nephrology fellows could perform renal biopsy safely under the supervision of an attending nephrologist [6,7]. Furthermore, not only supervision during the procedure, but also the education system for nephrology fellows may be important factors for the safety of the procedure by nephrology fellows. In the United States and Europe, the American Society of Nephrology and The Renal Section of the European Union of Medical Specialists developed curriculum for nephrology fellowship, respectively [17,18]. Physicians with short nephrology experience in this study were also training according to the curriculum at institutions certified by the Japanese Society of Nephrology. In summary, our results suggest that, even in the hands of physicians with little experience in clinical nephrology, renal biopsy can be taught and performed safely under the supervision of an attending nephrologist and a systematic nephrology training system.

All renal biopsies in our study were performed in an in-hospital setting and patients were ordered to strict bed rest for about 16 h following the procedure. The setting and post-biopsy rest time may vary from facility to facility. Since hospitalization and prolonged strict bed rest are difficult, some facilities perform outpatient renal biopsy with short bed rest durations. While the safety of this approach has been previously reported [19,20,21], long-term strict bed rest while hospitalized is likely to be safer because complications may occur up to 24 h after renal biopsy [4]. In addition, antiplatelet and anticoagulant drugs pose a risk of post-biopsy bleeding [22]. In this study, these drugs were stopped and a heparin bridge was performed in all patients receiving these medications. These stricter criteria might contribute to the safety of renal biopsy performed by physicians with short nephrology experience.

While safety is important, maintaining the diagnostic power of renal biopsy is equally critical. The glomerular sample size is a recognized indicator of the diagnostic power of renal biopsy. For light microscopic diagnosis, at least 8 to 10 glomeruli are needed [23,24]. Here, the mean number of glomeruli obtained by physicians with <6 months of experience in nephrology was 20.1 ± 11.6, which is sufficient for light microscopic diagnosis. This suggested that attending supervision ensures not only the safety but also the quality of renal biopsy.

This study has several limitations. First, this was a retrospective and non-randomized study; thus, a causal relationship between clinical nephrology experience and the safety of renal biopsy cannot be conclusively determined. Furthermore, the sample size of this study may be inadequate and we cannot completely exclude the possibility of type 2 error in the sample size setting. This study’s sample size was similar to those of previous reports investigating the safety of renal biopsy by nephrology fellows [6,7]. However, it may still be necessary to further conduct studies of a larger sample size to resolve this limitation. Second, this study was conducted in a single academic center in Japan. Thus, the results may not necessarily apply to all hospitals in Japan and other countries. However, as mentioned above, a systematic education system and supervision by an attending nephrologist are essential for the safety of renal biopsies performed by physicians with short nephrology clinical experience in not only Japan, but also other countries. Thus, under such conditions, our results could be applicable on a global scale. Third, we could not exclude the existence of unrecognized confounding factors that may affect the incidence of post-biopsy complications. Fourth, we could not collect some types of post-biopsy complications such as hematoma and macroscopic hematuria. Thus, our results may not apply to these types of complications.

## 5. Conclusions

Our study showed that under supervision, physicians with short clinical nephrology clinical experience can perform renal biopsy as effectively and safely as attending nephrologists. Such results suggest that nephrology trainees can be safely trained to perform renal biopsies with appropriate supervision.

## Figures and Tables

**Table 1:** Patient characteristics.

Characteristic	All Renal Biopsy (*n* = 238)
Age (years)	54.1 ± 18.1
Sex (Male:Female)	127:111
BMI (kg/m^2^)	22.7 ± 4.4
Hypertension (%)	50.6
Diabetes mellitus (%)	14.3
Systolic BP (mmHg)	128.2 ± 18.1
Diastolic BP (mmHg)	77.3 ± 13.0
Hb (g/dL)	12.2 ± 2.2
Plt (/μL)	25.7 ± 9.8
APTT (s)	28.5 ± 6.9
PT-INR	1.00 ± 0.12
Alb (g/dL)	3.4 ± 0.9
CRP (mg/dL)	0.10 (0.04–0.38)
eGFR (mL/min/1.73 m^2^)	54.9 ± 27.9
Urinary protein (g/gCr)	1.41 (0.55–4.16)
Length of biopsied kidney (cm)	10.0 ± 1.0
Number of punctures	3 (2–3)
Number of glomeruli	20.4 ± 11.1
Number of glomeruli <10 (%)	13.9
Post-biopsy Hb decline (g/dL)	0.3 ± 0.8
Post-biopsy Hb decline (%)	2.4 ± 7.0
Post-biopsy Hb decline ≥10% (%)	13.1
Post-biopsy hypotension (%)	3.8
Post-biopsy transfusion (%)	0.8
Post-biopsy renal artery embolism (%)	0
Post-biopsy nephrectomy (%)	0
Post-biopsy death (%)	0
Composite complication rate (%)	16.0
Number of physicians	18
Number of procedures per physician	13.2 ± 8.1

Alb, albumin; APTT, activated partial thromboplastin time; BMI, body mass index; BP, blood pressure; CRP, C-reactive protein; eGFR, estimated glomerular filtration rate; Hb, hemoglobin; Plt, platelet; PT-INR, prothrombin time international normalized ratio.

**Table 2:** Comparison of renal biopsies performed by physicians with <3 years and ≥3 years of clinical nephrology experience.

Characteristic	Physician with <3 Years of Clinical Nephrology Experience	Physician with ≥3 Years of Clinical Nephrology Experience	*p*-Value
	(*n* = 198)	(*n* = 40)	
Age (years)	53.7 ± 18.6	56.1 ± 15.7	0.61
Sex (Male:Female)	103:94	24:17	0.50
BMI (kg/m^2^)	22.8 ± 4.4	22.0 ± 4.3	0.38
Hypertension (%)	49.5	56.1	0.49
Diabetes mellitus (%)	14.8	12.2	0.81
Systolic BP (mmHg)	128.5 ± 18.0	126.6 ± 18.7	0.90
Diastolic BP (mmHg)	77.5 ± 13.4	76.4 ± 11.0	0.74
Hb (g/dL)	12.2 ± 2.1	11.7 ± 2.3	0.20
Plt (/μL)	26.0 ± 9.9	24.6 ± 9.4	0.52
APTT (s)	28.4 ± 7.3	29.4 ± 4.3	0.03
PT-INR	1.00 ± 0.12	1.03 ± 0.11	0.018
Alb (g/dL)	3.4 ± 0.9	3.2 ± 1.0	0.17
CRP (mg/dL)	0.07 (0.03–0.35)	0.21 (0.01–1.45)	<0.01
eGFR (mL/min/1.73 m^2^)	56.3 ± 28.6	48.3 ± 23.8	0.10
Urinary protein (g/gCr)	1.31 (0.50–3.82)	1.96 (0.89–5.94)	0.045
Length of biopsied kidney (cm)	10.0 ± 1.0	9.6 ± 1.1	0.06
Number of punctures	3 (2–3)	3 (2–3)	0.17
Number of glomeruli	20.3 ± 11.1	20.7 ± 11.2	0.90
Number of glomeruli <10 (%)	14.2	12.2	>0.99
Post-biopsy Hb decline (g/dL)	0.3 ± 0.8	0.3 ± 0.8	0.86
Post-biopsy Hb decline (%)	2.4 ± 6.9	2.2 ± 7.2	>0.99
Post-biopsy Hb decline ≥10% (%)	13.7	10.0	0.62
Post-biopsy hypotension (%)	4.6	2.6	>0.99
Post-biopsy transfusion (%)	0.5	2.6	0.31
Composite complication rate (%)	16.8	12.5	0.64
Number of physicians	14	4	
Number of procedures per physician	14.1 ± 8.5	10.3 ± 6.6	0.49

Alb, albumin; APTT, activated partial thromboplastin time; BMI, body mass index; BP, blood pressure; CRP, C-reactive protein; eGFR, estimated glomerular filtration rate; Hb, hemoglobin; Plt, platelet; PT-INR, prothrombin time international normalized ratio.

**Table 3:** Comparison of renal biopsies performed by physicians with <6 months and ≥6 months of clinical nephrology experience.

Characteristic	Physician with <6 Months of Clinical Nephrology Experience	Physician with ≥6 Months of Clinical Nephrology Experience	*p*-Value
	(*n* = 77)	(*n* = 161)	
Age (years)	52.4 ± 19.9	54.9 ± 17.2	0.44
Sex (Male:Female)	40:37	87:74	0.78
BMI (kg/m^2^)	23.1 ± 4.6	22.5 ± 4.3	0.40
Hypertension (%)	52.6	49.7	0.68
Diabetes mellitus (%)	13.7	14.9	0.84
Systolic BP (mmHg)	129.6 ± 16.8	127.5 ± 18.7	0.32
Diastolic BP (mmHg)	77.8 ± 12.4	77.1 ± 13.3	0.60
Hb (g/dL)	12.5 ± 2.3	12.0 ± 2.1	0.12
Plt (/μL)	25.1 ± 8.3	26.1 ± 10.5	0.56
APTT (s)	28.5 ± 7.8	28.6 ± 6.4	0.88
PT-INR	1.01 ± 0.14	1.00 ± 0.10	0.68
Alb (g/dL)	3.4 ± 1.0	3.4 ± 0.9	0.84
CRP (mg/dL)	0.07 (0.03–0.30)	0.13 (0.04–0.47)	0.16
eGFR (mL/min/1.73 m^2^)	56.4 ± 27.8	54.2 ± 28.0	0.64
Urinary protein (g/gCr)	1.11 (0.44–4.20)	1.58 (0.61–4.16)	0.22
Length of biopsied kidney (cm)	9.8 ± 0.9	10.0 ± 1.1	0.26
Number of punctures	2 (2–3)	3 (2–3)	0.26
Number of glomeruli	20.1 ± 11.6	20.5 ± 10.9	0.62
Number of glomeruli <10 (%)	14.3	13.7	>0.99
Post-biopsy Hb decline (g/dL)	0.3 ± 0.9	0.3 ± 0.8	0.70
Post-biopsy Hb decline (%)	2.6 ± 7.3	2.3 ± 6.8	0.58
Post-biopsy Hb decline ≥10% (%)	11.7	13.8	0.84
Post-biopsy hypotension (%)	7.8	2.5	0.08
Post-biopsy transfusion (%)	0	1.3	>0.99
Composite complication rate (%)	15.6	16.3	>0.99
Number of physicians	13	14	
Number of procedures per physician	5.9 ± 3.1	11.5 ± 2.3	0.02

Alb, albumin; APTT, activated partial thromboplastin time; BMI, body mass index; BP, blood pressure; CRP, C-reactive protein; eGFR, estimated glomerular filtration rate; Hb, hemoglobin; Plt, platelet; PT-INR, prothrombin time international normalized ratio.

**Table 4:** Logistic regression analysis for risk of composite complication after renal biopsy.

Characteristic	Univariate			Multivariate			Multivariate		
				(Model 1)			(Model 2)		
	OR	95% CI	*p*-Value	OR	95% CI	*p*-Value	OR	95% CI	*p*-Value
Age (years)	1.00	0.98–1.02	0.62	1.00	0.98–1.03	0.83	1.00	0.98–1.03	0.84
Male	1.70	0.84–3.40	0.14	1.68	0.80–3.54	0.17	1.69	0.80–3.55	0.17
Hb (g/dL)	1.00	0.85–1.18	0.98	0.99	0.82–1.20	0.95	0.99	0.82–1.20	0.91
eGFR (mL/min/1.73 m^2^)	1.00	0.99–1.01	0.84	1.00	0.99–1.02	0.87	1.00	0.99–1.02	0.90
Physician with <6 months of clinical nephrology experience	1.05	0.50–2.21	0.89	1.08	0.51–2.30	0.84			
Physician with <3 years of clinical nephrology experience	0.71	0.26–1.95	0.51				0.75	0.27–2.08	0.58

CI, confidence interval; eGFR, estimated glomerular filtration rate; Hb, hemoglobin; OR, odds ratio.

## Data Availability

The data presented in this study are available on request from the corresponding author.
